# An Important Invention

**Published:** 1870-01

**Authors:** 


### AN IMPORTANT INVENTION.

We have just examined an invention of a professional brother. Dr. W. P. Hall, of Piqua, Ohio, which consists of a smoke consuming stove, for bituminous coal. It also burns equally well coke or anthracite coal; but the great aim in the invention is the consumption of all the combustible material of soft coal, and by this invention it is perfectly accomplished. The object is effected by causing the gas and carbon that is evolved from the burning coal to pass through a bed of burning coke and at the same time supplying the necessary oxygen from the air thrown into the burning coke.

This principle, as thus developed, must work an entire revolution wherever bituminous coal is used. Away with all the smokey, sooty cities. No excuse for them now.

The stoves are in form not like anything else we have ever seen. They are ornamental—may be made very beautiful. They will give more heat than any other stove of the same size.

From a given quantity of coal more than twice the amount of heat is obtained than when burned in an ordinary grate, and quite 50 per cent. more than when burned in an ordinary stove. It is either a close stove or an open grate. It may be used in any place where heat is required. T.

